# Effects of Various Antibiotics Alone or in Combination with Doripenem against *Klebsiella pneumoniae* Strains Isolated in an Intensive Care Unit

**DOI:** 10.1155/2014/397421

**Published:** 2014-10-28

**Authors:** Berna Ozbek Celik, Emel Mataraci-Kara, Mesut Yilmaz

**Affiliations:** ^1^Department of Pharmaceutical Microbiology, Faculty of Pharmacy, Istanbul University, Beyazit, 34116 Istanbul, Turkey; ^2^Department of Infectious Diseases and Clinical Microbiology, Faculty of Medicine, University of Istanbul Medipol, 34083 Istanbul, Turkey

## Abstract

Colistin, tigecycline, levofloxacin, tobramycin, and rifampin alone and in combination with doripenem were investigated for their in vitro activities and postantibiotic effects (PAEs) on *Klebsiella pneumoniae*. The in vitro activities of tested antibiotics in combination with doripenem were determined using a microbroth checkerboard technique. To determine the PAEs, *K. pneumoniae* strains in the logarithmic phase of growth were exposed for 1 h to antibiotics, alone and in combination. Recovery periods of test cultures were evaluated using viable counting after centrifugation. Colistin, tobramycin, and levofloxacin produced strong PAEs ranging from 2.71 to 4.23 h, from 1.31 to 3.82 h, and from 1.35 to 4.72, respectively, in a concentration-dependent manner. Tigecycline and rifampin displayed modest PAEs ranging from 1.18 h to 1.55 h and 0.92 to 1.19, respectively. Because it is a beta-lactam, PAEs were not exactly induced by doripenem (ranging from 0.10 to 0.18 h). In combination, doripenem scarcely changed the duration of PAE of each tested antibiotic alone. The findings of this study may have important implications for the timing of doses during *K. pneumoniae* therapy with tested antibiotics.

## 1. Introduction

*Klebsiella pneumoniae* can cause different types of healthcare-associated infections, including pneumonia, bloodstream infections, wound or surgical site infections, and meningitis [1]. In healthcare settings, patients whose care requires devices like ventilators or intravenous catheters and patients taking long courses of certain antibiotics are most at risk for* Klebsiella* infections [1, 2]. Because* K. pneumoniae* has the ability to acquire resistance against different classes of antibiotics, including carbapenems [3], it has been an important consideration in the development of effective combination therapy, both to rapidly enhance bactericidal activity and to help prevent or delay the emergence of resistance [4, 5]. Studies have proven that combinations of a doripenem with colistin or tigecycline or tobramycin or levofloxacin or rifampin can produce synergy [6–11]. Additionally, optimization of dosing by pharmacodynamic parameters has also been shown to improve outcomes of* K. pneumoniae* infections [12, 13].

PAE, a pharmacodynamic parameter, is defined as the suppression of bacterial growth observed after removal of an antimicrobial agent from the culture medium [14–16]. Additionally, extending the dosing interval of an antimicrobial agent with a PAE has potential advantages: for example, reduced cost and less toxicity [17]. PAE can be used to develop more effective dosing regimens to improve the efficiency of antimicrobial agents, reduce the emergence of resistance, and develop new drugs and new formulations and should be considered during guideline formation and development [18].

According to our research, a limited number of reports addressing the PAE of antibiotics have been published regarding* K. pneumonia*. Therefore, the present study aimed to identify the PAE interaction of the different groups of antibiotics alone or in combination with doripenem against* K. pneumoniae* strains isolated from bloodstream infections. Furthermore, we investigated whether PAEs induced by the tested antibiotic combinations differ from those induced by colistin, tigecycline, levofloxacin, tobramycin, or rifampicin alone for* K. pneumoniae* strains.

## 2. Materials and Methods

### 2.1. Bacterial Isolates

Six nonduplicate, nosocomially acquired* K. pneumoniae* strains isolated from blood specimens between January and June 2011 were obtained from the Department of Infectious Diseases and Clinical Microbiology, Faculty of Cerrahpasa Medicine, Istanbul University. All strains were identified using API 20NE (bioMérieux). As a reference strain,* K. pneumonia* ATCC 700603 (American Type Culture Collection, Rockville, MD, USA) was used throughout the study to verify the accuracy of microdilution test procedure to ensure that MIC values of the antibiotics studied were within the accuracy range stated by the Clinical and Laboratory Standards Institute (CLSI) [19].

### 2.2. Antibiotics

All antimicrobial agents were kindly provided by their respective manufacturers. Stock solutions of colistin sulphate, tobramycin, levofloxacin, and rifampin were stored frozen at −80°C. Frozen solutions of antibiotics were used within six months. Tigecycline and doripenem solutions were prepared on the day of use.

### 2.3. Media

Mueller-Hinton broth (Difco Laboratories, Detroit, MI, USA) was used for MIC, and PAE studies, and supplemented with 25 mg of calcium/liter and 12.5 mg of magnesium/liter (CAMHB). The broth was used within 24 h of preparation for the tigecycline [20]. Pour plates of tryptic soy agar (Difco Laboratories) were used for colony counts.

### 2.4. MIC Determinations

MICs were determined by the microbroth dilution technique described by CLSI. Serial twofold dilutions ranging from 512 to 0.250 mg/L for rifampin, from 128 to 0.06 mg/L for doripenem, tobramycin, levofloxacin, and from 32 to 0.015 mg/L for tigecycline and colistin were prepared in fresh CSMHB 96-well microtiter plates. The inoculum was prepared with a 4- to 6-h broth culture. Each isolate was adjusted spectrophotometrically to 1 × 10^8^ CFU/mL (OD_600_ 0.12-0.13) and diluted in CSMHB to create a final concentration of 5 × 10^5^ CFU/mL in the test tray. The trays were covered, placed in plastic bags to prevent evaporation, and incubated at 37°C for 18–20 h. The MIC was defined as the lowest concentration of antibiotic giving complete inhibition of visible growth.

### 2.5. Determination of the Fractional Inhibitory Concentration Index

The effects of antibiotics in combination were assessed using a microbroth checkerboard technique [21]. Each microtiter well containing the mixture of antibiotics was inoculated with a 4- to 6-hour broth culture diluted to produce a final concentration of approximately 5 × 10^5^ CFU/mL. After incubation at 37°C for 18–20 h, the fractional inhibitory concentration (FIC) index was determined as the combined concentration divided by the single concentration. The combination value was derived from the highest dilution of antibiotic combination permitting no visible growth. With this method, synergy was defined as an FIC index ≤0.5, no interaction as an FIC index between 0.5 and 4, and antagonism as an FIC index ≥4.0 [22].

### 2.6. Determination of PAE

PAEs were determined by a standard viable counting method [15]. Samples were incubated for 1 h to avoid prolonged antibiotic exposure and consequent complete eradication of the organism. At time zero, 1 mL of inoculum was added to tubes containing 29 mL CSMHB with or without test antibiotics. Organisms in the logarithmic phase of growth, producing a final concentration of inoculums in the test tubes of approximately 10^6^ CFU/mL, were exposed to concentrations of doripenem, tigecycline, colistin, levofloxacin, tobramycin, and rifampin equal to 1x MIC or 4x MIC, alone or in combination. After incubation for 1 h in a 37°C shaking water bath, antibiotics were removed by centrifugation at 5,000 rpm for 10 min. Then supernatant was decanted and cells were washed twice in buffered sterile saline (0.9% NaCl) before being resuspendedin 30 mL of prewarmed CSMHB. Bacterial counts of tube contents were determined at time zero, immediately before and after centrifugation, and each h after centrifugation for 8 h by spreading on pour platesusing 10-fold dilutions in cold saline as required. Antimicrobial carryover was controlled by the inhibition of colonial growth at the site of the initial streak according to NCCLS guidelines [23].

Plates were read after incubation for 18–24 h at 37°C. The PAE was defined according to Craig and Gudmundsson [Craig] as PAE = *T* − *C*, where *T* is the time (in h) required for the viability count in the test culture to increase 1 log⁡_10_⁡ above the count observed immediately after centrifugation, and *C* is the corresponding time for the controls. Experiments were conducted in triplicate.

### 2.7. Statistical Analysis

Statistical analysis was done with GraphPad Prism 5.0 (GraphPad Software Inc., San Diego, CA, USA). Results are expressed as mean ± SD. One-way ANOVA followed by Bonferroni's multiple comparison test was performed to examine the change in PAE values of each antibiotic concentration alone and in combination. In the results, alpha < 0.05 was considered significant.

## 3. Results

The MICs of doripenem, colistin, tobramycin, levofloxacin, tigecycline, and rifampin against six tested clinical strains and the reference strains of* K. pneumoniae* ATCC 700603 are shown in Table 1. With an FIC index ≤0.5 as the borderline value, synergy was detected against 1 strain with the doripenem-tigecycline and the doripenem-colistin combination, and against 2 strains with the doripenem-levofloxacin and doripenem-tobramycin combinations (Table 1). Antagonism was not observed with any combination.

**Figure 1:**
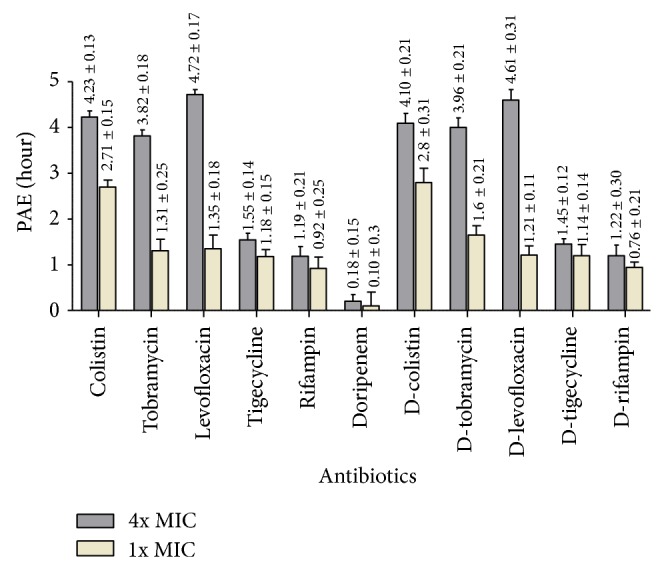
The mean PAE values for six clinical strains of* K. pneumonia*.

The mean PAE values for six clinical strains of* K. pneumoniae* are displayed in Figure 1. For a concentration of 1x MIC, colistin, tobramycin, and levofloxacin showed a good PAE for all strains, varying between 2.34 and 3.13 h, between 1.01 and 1.59 h, and between 1.05 to 1.56 h, respectively. Tigecycline and rifampin showed a modest PAE for all strains, varying between 0.95 and 1.39 h and between 0.73 and 1.37 h, respectively. When the concentrations of tested antibiotics were increased to 4x MIC, the duration of the PAEs were significantly prolonged: colistin produced PAEs ranging from 3.92 to 4.51 h (*P* < 0.05); levofloxacin produced PAEs ranging from 4.42 to 4.92 h (*P* < 0.0001); and tobramycin produced PAEs ranging from 3.56 to 4.16 h (*P* < 0.001). Although tigecycline and rifampin at 4x MIC produced PAEs from 1.38 to 1.77 and from 0.92 to 1.45, respectively, in a concentration-dependent manner, no statistically significant difference was found (*P* > 0.05). On the other hand, negligible PAE values were obtained with doripenem at both 1x MIC or 4x MIC against the studied strains. As seen in Figure 1, doripenem combined with the tested antibiotics at concentrations of 1x MIC or 4x MIC did not produce significantly different PAEs than when the antibiotics were used alone (*P* > 0.05).

## 4. Discussion

Determination of the postantibiotic effects is an important part of preclinical evaluation of antibiotics because it is a factor that influences antibiotic dosing intervals [36–38]. PAE is likely the result of several mechanisms, including nonlethal damage caused by the antibiotic and continued persistence of the drug at the bacterial drug-binding site for a given period of time after the drug is removed [39]. For example, recovery from the postantibiotic effect induced by tobramycin in* Escherichia coli* depends upon reestablishment of protein synthesis, and recovery from the levofloxacin-induced postantibiotic effect depends upon restoration of DNA synthesis [33].

In the present study, PAEs of all tested antibiotics were determined and compared with the previous studies in Table 2. Fluoroquinolones generally produce PAEs against Gram-negative and positive strains [24, 25, 29, 40, 41]. Consistent with previous study by Spangler et al. [24] our results display that levofloxacin possesses strong PAE values against* K. pneumoniae* strains. This antibiotic at 4x MIC concentrations alone exhibited the most prolonged PAEs compared to all tested antibiotics. Since the clinical implication of long PAEs lies in the possibility of increasing the intervals between drug administrations, thus allowing for fewer daily dosages and thereby potentially reducing treatment costs, increasing patient compliance and decreasing drug exposure [42], administering once-daily levofloxacin might be advantageous for patient outcomes.

The results of this research also indicate that colistin has potent PAEs against the tested* K. pneumoniae* strains. Similar to a previous study [25–28], in the present study this antibiotic displayed powerful PAEs: at 4x MIC alone, it exhibited nearly twice as long PAEs than when the antibiotic was used at 1x MIC against the tested strains. However, a recent increase in the prevalence of multidrug resistant* K. pneumoniae* and the lack of novel agents in development calls for a need to reexamine the colistin therapy.

On the other hand, the present investigation showed that tobramycin has significant PAEs against the tested six* K. pneumonia* strains. This is in agreement with the previous results [17, 29, 30]. This antibiotic at 4x MIC alone prolonged PAEs by more than three times than when the antibiotic was used at 1x MIC concentrations. The benefit of this prolonged PAE value of tobramycin may allow for prolonged dose intervals without reduced efficacy and possibly a lower frequency of adverse events during* K. pneumoniae* therapy.

A limited number of reports addressing tigecycline PAE have been published focusing on* K. pneumoniae* [31, 32]. All these studies and ours have shown that the activity of tigecycline's PAE, which has also been evaluated both in vitro and in vivo, is good and changes with increasing concentrations.

Inter alia, antibiotic combinations which include rifampin may have a role in the treatment of* K. pneumoniae* and possibly slow the selection of heteroresistant subpopulations during therapy [11, 43]. According to PAE studies on rifampin, this antibiotic induced a postantibiotic effect against* E. coli* [33] and* Legionella* spp. [34]. So far, ours is the first study in which a PAE for rifampin has been clearly demonstrated on* K. pneumoniae*. In the present study, a moderate PAE was produced by rifampin both at 1x MIC and at 4x MIC against the studied strains.

Lastly, very negligible PAE was produced by doripenem at 1x MIC or 4x MIC against the studied strains. Although there is no prior publication on the PAEs of doripenem against* K. pneumoniae,* our results were aligned with similar results of previous studies suggesting that PAEs have been described for wide variety of antibiotics used singly against Gram-negative strains, but only for non-beta-lactams, with the exception of carbapenems on* P. aeruginosa* [35, 44, 45].

The increasing interest in combination therapy for* K. pneumoniae* infections is mostly due to the organism's ability to acquire resistance against different classes of antibiotics, including carbapenems, with a limited availability of effective agents [3]. In our in vitro study, synergistic activity for each combination was seen at least against one clinical strain, except for doripenem-rifampin.

The other purpose of this study was to examine whether PAEs induced by drug combinations differed from PAEs induced by the drugs alone. The tested combinations produced similar PAEs from the PAEs induced by the colistin or tobramycin or levofloxacin or tigecycline or rifampin alone; statistically significant differences in PAEs were not determined, comparatively (*P* > 0.05).

Consequently, the main findings of this study are that PAE values of tested antibiotics (except for doripenem), levofloxacin, colistin, tobramycin, tigecycline, and rifampicin, have an ability to produce PAEs against* K. pneumoniae* and may have important implications for the dosing regimen treatment of* K. pneumoniae* infections. Also, PAEs induced by drug combinations were not different from PAEs induced by the drugs alone.

## Figures and Tables

**Table 1:** In vitro activities of antibiotics alone (MIC, mg/L) and in combination (FIC index) with doripenem against the studied strains.

	KP-700606	KP-1	KP-2	KP-3	KP-4	KP-5	KP-6
Antibiotic							
DOR	0.06	16	16	16	16	16	16
CS	1	0.5	0.25	1	1	1	1
TGC	0.12	0.25	0.125	0.125	0.06	0.25	0.125
TOB	0.5	2	1	2	1	0.5	1
LVX	0.06	1	0.03	0.06	0.125	0.5	2
RIF	512	256	256	512	256	512	512
Combination							
DOR + TGC	0.75	1	0.75	0.75	0.25	1	0.75
DOR + CS	0.75	1	0.5	1	0.75	1	0.75
DOR + LVX	0.5	1	0.5	1	0.125	0.75	1
DOR + TOB	1	0.25	1	1	0.25	1	1
DOR + RIF	1	1	0.75	1	2	1	0.75

DOR: doripenem; TGC: tigecycline; CS: colistin sulphate; LVX: levofloxacin; TOB: tobramycin; RIF: rifampin. KP-700606: reference strain (*Klebsiella pneumoniae *ATCC 700606); KP: *Klebsiella pneumoniae*.

**Table 2:** The PAE values for tested antibiotics.

Antibiotic	Bacteria	Special conditions	Duration of PAE (H)	Author/year	Reference number
Levofloxacin	*K. pneumoniae *	1 and 4x MIC	1.35 and 4.72	Present study	
	*K. pneumoniae *	0.5x MIC	1.80	Spangler et al./2000	[24]

Colistin	*K. pneumoniae *	1 and 4x MIC	2.71 and 4.23	Present study	
	*A. baumannii *	1 and 4x MIC	3.00 and 6.28	Özbek and Şentürk/2010	[25]
	*P. aeruginosa *	1 and 20x MIC	1.13 and 2.12	Bozkurt-Güzel and Gerçeker/2012	[26]
	*P. aeruginosa *	16x MIC	2.00 and 3.00	Li et al./2001	[27]
	*A. baumannii* ATCC 19606	16, 32 and 64x MIC	1.00, 2.30 and 3.50	Owen et al./2007	[28]

Tobramycin	*K. pneumoniae *	1 and 4x MIC	1.31 and 3.82	Present study	
	*P. aeruginosa *	1 and 10x MIC	1.50 and 3.10	Ozbek and Otuk/2009	[29]
	*P. aeruginosa *	In vivo	2.00 to 4.00	Gudmundsson et al./1993	[30]
	Gram-negative bacteria	In vivo	3.00 to 4.00	Spivey/1992	[17]

Tigecycline	*K. pneumoniae *	1 and 4x MIC	1.18 and 1.55	Present study	
	*K. pneumoniae *	2 and 10x MIC	1.70 and 1.80	Pankuch and Appelbaum/2009	[31]
	*Enterococcus faecalis *	1 to 20x MIC	1.00 and 4.50	Lefort et al./2003	[32]

Rifampin	*K. pneumoniae *	1 and 4x MIC	0.92 and 1.19	Present study	
	*E. coli *ATCC 25922	5x MIC	4.00	Stubbings et al./2006	[33]
	*Legionella pneumophila *	4x MIC	2.86 and 3.09	Dubois and St-Pierre/2000	[34]

Doripenem	*K. pneumoniae *	1 and 4x MIC	0.10 and 0.18	Present study	
	*E. coli and P. aeruginosa *	10x MIC	Weak or No PAE for meropenem	Odenholt-Tornqvist/1993	[35]
